# Induction of chromosome instability and stomach cancer by altering the expression pattern of mitotic checkpoint genes in mice exposed to areca-nut

**DOI:** 10.1186/1471-2407-13-315

**Published:** 2013-06-28

**Authors:** Sillarine Kurkalang, Atanu Banerjee, Nitin Ghoshal, Hughbert Dkhar, Anupam Chatterjee

**Affiliations:** 1 Molecular Genetics Laboratory, Department of Biotechnology & Bioinformatics, North-Eastern Hill University, Shillong, Meghalaya 793022, India; 2 Histopathology Division, Nazareth Hospital, Laitumkhrah, Shillong 793003, India

**Keywords:** Chromosome instability, Mitotic checkpoint genes, *Securin*, Apoptosis

## Abstract

**Background:**

There are strong indications for a causal association between areca-nut consumption and cancers. In Meghalaya, India, the variety of areca-nut is used as raw and unprocessed form whose chemical composition and pharmacological actions have been reported. Yet we know little on the initial pathway involved in areca-nut associated carcinogenesis since it is difficult to assess its effects on genetic alterations without interference of other compounding factors. Therefore, present study was undertaken in mice to verify the ability of raw areca-nut (RAN) to induce cancer and to monitor the expression of certain genes involved in carcinogenesis. This study was not intended to isolate any active ingredients from the RAN and to look its action.

**Methods:**

Three groups of mice (n = 25 in each) were taken and used at different time-points for different experimental analysis. The other three groups of mice (n = 15 in each) were considered for tumor induction studies. In each set, two groups were administered RAN-extract *ad libitum* in drinking water with or without lime. The expression of certain genes was assessed by conventional RT-PCR and immunoblotting. The mice were given the whole RAN-extract with and without lime in order to mimic the human consumption style of RAN.

**Results:**

Histological preparation of stomach tissue revealed that RAN induced stomach cancer. A gradual increase in the frequency of precocious anaphase and aneuploid cells was observed in the bone marrow cells with a greater increment following RAN + lime administeration. Levels of *p53, Bax, Securin* and *p65* in esophageal and stomach cells were elevated during early days of RAN exposure while those of different mitotic checkpoint proteins were downregulated. Apoptotic cell death was detected in non-cancerous stomach cells but not in tumor cells which showed overexpression of *Bax* and absence of *PARP*.

**Conclusion:**

Present study suggested (a) RAN induces stomach cancer, however, presence of lime promoted higher cell transformation and thereby developed cancer earlier, (b) perturbations in components of the chromosome segregation machinery could be involved in the initial process of carcinogenicity and (c) the importance of precocious anaphase as a screening marker for identification of mitotic checkpoint defects during early days.

## Background

Esophageal squamous cell carcinoma (ESCC) and the gastric cancers are most common cancers in India, with the highest incidence of ESCC being in north-eastern states of India [1]. There are strong indications for a causal association between areca-nut or quid chewing habits and these cancers. Several studies in different animal species have shown positive induction of tumors in both target (cheek-pouch, esophagus and stomach) and non-target (lung and liver) tissues when arecoline (ARC) or areca-nut extract was administered by different means such as oral intubation [2], mixed with the diet [3], and cheek-pouch application [4]. Therefore, it seems that metabolic activation of alkaloids is needed for the final conversion into the ultimate carcinogens, which is strongly influenced by physiological conditions and presence of certain factors [5]. Reports have indicated generation of reactive oxygen species (ROS) from areca-nut ingredients under alkaline conditions [6,7]. Due to the presence of lime in betel-quid preparation, areca-nut chewers’ saliva typically changes from neutral to an alkaline condition which could be ideal for generating ROS [7]. Nair et al. [6] have also noted that besides ARC, auto-oxidation of areca-nut polyphenols could generate H_2_O_2_ and superoxide radicals at alkaline pH.

In the State of Meghalaya, India, the variety of areca nut, locally called ‘kwai’, is used as unripe and unprocessed raw form which has higher contents of alkaloids, polyphenols and tannins as compared to the dried form [8]. The betel-quid used in Meghalaya contains raw areca-nut (RAN), lime paste and small portion of betel-leaf without tobacco and other constituents. Here people swallow the whole quid after chewing instead of spitting it out which could be an important factor for ESCC and stomach cancer. Recently, 40% esophageal cancer samples collected from patients of Meghalaya state having only RAN-chewing habit showed deletion of the microsatellite markers D9S1748 and D9S1749, located close to exon 1β of *CDKN2A/ARF* gene at 9p21. The promoter hypermethylation of *CDKN2A* gene was significantly higher in the samples with the habit of RAN-chewing alone than those having the habit of use both RAN and tobacco [9]. Till now, we do not know much on the initial pathway involves in betel-nut associated carcinogenesis in esophagus and stomach. It is also difficult to assess the effects of purely and predominantly areca-nut-induced genetic alterations in human without interference by other compounding factors like tobacco chewing or smoking, alcohol consumption, various types of non-vegeterian foods etc. Moreover, the presence of lime makes an alkaline condition which is not ideal for *in vitro* cell culture and therefore the effect of areca-nut cannot be tested in cell culture systems. In view of these, the present study was carried out in mice to verify the ability of RAN-extract with or without lime, to induce cancer and simultaneously evaluate the expression pattern of certain genes which play an important role in the initial process of carcinogenesis.

The chemical composition and pharmacological actions of areca-nut have been reported and reviewed by several workers [10,11]. Several animal studies have confirmed that areca-nut products and betel specific nitrosamines, have the ability to induce neoplastic changes in experimental animals [11]. Considerable evidence suggests that areca-nut-alkaloids, predominantly arecoline (ARC) are the major factors in BN-toxicity [11]. It was shown that ARC can induce DNA damages in mouse bone marrow cells [12] and such DNA damages can be reduced when ARC is administered with N-acetyl-L-cysteine [13]. Therefore, it is worth mentioning that the present study was not intended to isolate any active ingredients from the RAN and to look its action. The aim of the study was to identify the initial pathway involved in RAN associated carcinogenesis in mice and therefore mice were given the whole RAN-extract with and without lime in order to mimic the consumption habit of human.

Several genes, like *p53, p65, Securin* and many others, are known to be usually overexpressed during carcinogenesis [14-16]. Moreover, genetic instability is also associated with chromosome instability (CIN) which leads to aneuploidy, a hallmark of cancer. Such aneuploidy may facilitate tumorigenesis through the loss of tumor suppressor gene function. It has been observed that the partial loss of mitotic checkpoint control leads to CIN in human cancer cells [17,18]. Therefore, in the present study, we evaluated the expression pattern of *p53, p65, Securin* and several mitotic and spindle assembly checkpoint genes at different time-points. We observed that RAN can induce stomach cancer by perturbing the components of the chromosome segregation machinery.

## Methods

### Preparation of extracts

After shelling the fibrous coats from unprocessed raw and unripe areca-nut (RAN), 100 g of RAN were ground and suspended in 125 ml of distilled water and mixed thoroughly to give a smooth paste for preparation of an aqueous extract of RAN. After 24 h, the paste was stirred for 3 h at 37°C and the aqueous extract was collected by centrifugation. This extraction procedure was repeated once more by adding 125 ml of water to the residue. Both extracts were pooled, representing 100 g of RAN in 250 ml distilled water, filtered and frozen at - 80°C. The filtrate was lyophilized in a Secfroid Lyolab BII Lyophilizer (Denmark). The lyophilized mass was kept at 4°C until use. The extract contained 0.9 g/100 g water-extractable material.

### Animals maintenance and treatment

Swiss albino mice (25–30 gm), 2–3 months old were maintained in the laboratory in community cages in a room with controlled temperature (20 ± 2°C) and controlled lighting (12 h light; 12 h dark). Standard mouse diet (NMC Oil Mills Ltd., Pune, India) and water *ad libitum* were used in all experiments. The experiments were conducted in compliance with institutional guidelines and approved by our “Institutional Standards for Animal Care and Use” Board.

**Figure 1:**
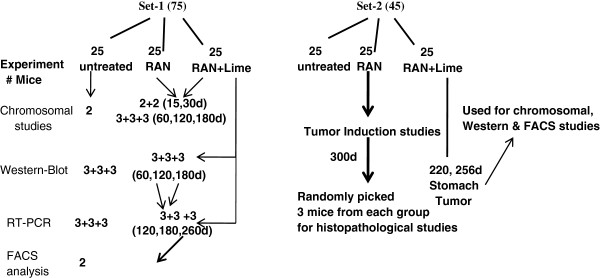
Flow diagram of experimental design for the analysis of raw areca-nut mediated Carcinogenesis in mice. RAN- raw areca-nut; d- days.

In Set-1, three groups of mice (n = 25 in each) were taken which were used at different time-points for different experimental analysis. In Set-2, three groups of mice (n = 15 in each) were considered for tumor induction studies. Figure 1 gives a schematic overview about the overall experimental protocol which was considered in this study. In each set, one group was treated with simple drinking water considered to be untreated whereas two groups were administered RAN extract *ad libitum* in the drinking water with or without lime (pH 9.8). It was estimated that each mouse consumed 1 mg of extract per day. Such oral administeration was continued for 60 days after which the dose was increased from 1 mg to 2 mg per day till 120 days. Likewise, every 60 days later the dose was increased by 1 mg per day consumption.

### Preparation of metaphases and scoring of chromosomal aberrations

For metaphase preparation, bone marrow cells (BMC) were collected from two mice per point from untreated, 15 and 30 days of treated group and three mice per point for the rest. In the treated groups, BMC were collected after 15, 30, 60, 120 and 180 days of treatment. BMC were also collected from the two mice having stomach tumor. BMC were collected after 2 h colchicine treatment (15 mg/kg). Animals were killed by cervical dislocation. The femurs were dissected out and the BMC were flushed out by injecting 2 ml 0.075 M KCl prewarmed to 37°C. Cells were treated in hypotonic solution for 15 min and fixed in acetic acid and methanol (1:3). Slides were prepared by the flame drying method, stained in 5% Giemsa for 5 min and mounted in synthetic medium. Images of metaphase spreads were taken under Zeiss Axioskop microscope (Germany).

For chromosomal study, the slides were coded at random and at least 100 well spread metaphase plates were selected for study from each mouse. We performed chromosome counts on metaphase spread. Chromosome aberrations were scored as isochromatid breaks and chromatid breaks. See Extended Experimental Procedures for details in the Additional file 1: Supplemental Information.

### Immunoblotting

Cells from bone-marrow, esophagus (by scratching inner layer) and stomach (by scratching inner part) were washed twice with PBS (phosphate buffered saline) and were lysed in radioimmuno-precipitation buffer (0.1% SDS, 2 mM EDTA, 1% NP-40, 1% sodium deoxycholate, 50 mM sodium fluoride and 100 U/ml aprotinin). After 30 min of incubation on ice, the cell lysates were centrifuged for 15 min at 4°C and the amount of protein was determined using the bicinchoninic acid protein assay. Equal amount of protein (40 μg) from each sample was loaded in each well; equal loading was further verified by immunoblotting with actin antibodies. Samples were loaded in Novex Tris-Glycine 4-20% gradient gels and electrophoresis was performed in NuPAGE electrophoresis system (Invitrogen, USA). Proteins were transferred to a Polyvinylidene difluoride (PVDF) membrane (Sigma) following standard protocol. The membranes were probed with a 1:1000 dilution of a mouse monoclonal antibody against *p53* (PAb 240; ab-26; Abcam, USA), *Bax* (6A7; ab5714; Abcam, USA), *Securin* (DCS-280; ab3305; Abcam, USA), *β-actin* (AC-15; ab6276; Abcam, USA) and rabbit polyclonal antibody against *NF-κβ P65* (ab31481; bcam, USA). Blots were washed 3 times for 10 min each in TBST buffer pH 7.6 (1 M Tris Cl, 5 M NaCl and 0.05% Tween 20) and incubated with secondary antibody (alkaline–phosphatase conjugated anti-mouse IgG or alkaline–phosphatase conjugated antirabbit IgG 1:2000; Abcam, USA) for 1 h at room temperature. After extensive washing, the blot was immersed in 4 ml substrate solution of BCIP/NBT (Bangalore Genei, India). Sufficient staining was obtained within 15 min. Each immunoblotting was performed in three mice per-point.

### Histopathological evaluation

Stomach tissue was collected from untreated control and from two RAN + lime treated mice with tumor. In another set, stomach tissue was also collected from untreated as well as from the groups that treated for 300 days with RAN-extract with and without lime. Three mice were selected randomly from each group. These mice did not have any indication of tumor externally. Tissue sections (5–7 μm) were processed for histological sectioning as per standard protocol [19] and stained with hematoxylin and eosin [20]. Sections were then observed under a light microscope and photographed (Carl Zeiss, Germany).

### RNA extraction and conventional RT-PCR analysis

Cells were collected by scratching the inner layer of esophagus and stomach from untreated control, RAN and RAN + lime treated mouse (three mice per point). Bone marrow cells were collected from the femur bone of the mouse. Total RNA was isolated with Trizol and then purified using the RNeasy Mini Kit (Qiagen) according to the manufacturer’s protocol. Reverse transcription was performed with 1 μg of total RNA from each sample using Quantiscript Reverse Transcriptase, Quantiscript RT-buffer and RT Primer-mix of QuantiTect Reverse Transcription kit (Qiagen GmbH, Hilden, Germany) according to the manufacturer's protocol. Amplification of cDNA was conducted in 20 μl solution containing 2 μl cDNA, 10 pmol primer pairs for aurora A, aurora B, Mad2, Bub1 and GAPDH (for primer sequences, see Additional file 1: Supplemental Information) respectively, and 10 μl of RT qPCR Master mix (Qiagen GmbH, Hilden, Germany). The PCR consisted of initial denaturation at 94°C for 5 min, followed by 30 reaction cycles (30 seconds at 94°C, 30 seconds at 60°C, and 30 seconds at 72°C) and a final cycle at 72°C for 10 min. GAPDH was used as internal control. All PCR products were electrophoretically separated on ethidium bromide-stained agarose gel and visualized with UV light.

### Flow cytometric analysis of cells

Mouse bone marrow cells and the cells collected by scratching the inner layer of the esophagus and stomach of both untreated and treated for 260 days with RAN with or without lime were fixed with 70% ethanol. Stomach tumor cells were also collected and fixed. The fixed cells were washed in PBS and resuspended in 500 μl of propidium iodide solution (50 μg/ml propidium iodide, 0.2 mg/ml RNase) for 1 h at room temperature in dark. 10,000 cells were acquired for each sample and analysed with a FACS Calibur (Becton-Dickinson). CELLQuest Pro software was used to quantify cell cycle compartments to estimate the percentage of cells distributed in the different cell cycle phases.

### Annexin V labelling studies

Apoptotic cell death was evaluated using annexinV–fluorescein isothiocyanate method in the stomach tumor cells and also in the inner layer of cells of the stomach and esophagus of untreated and RAN with and without lime treated mouse after 260 days of continuous administeration. The cell pellet was resuspended in PBS. Cells were stained with propidium iodide and Annexin-V-FITC using BD PharmingenTM Annexin V: FITC Apoptosis Detection Kit (BD-Pharmingen Biosciences, San Diego, CA) as per manufacturer’s instruction. Briefly, after collecting and washing twice with PBS, cells were resuspended in the binding buffer (500 μl). FITC-Annexin-V (5 μl) was added to the cells followed by addition of 5 μl PI according to the protocol. The samples were then incubated for 15 min in the dark at room temperature and subjected to flow cytometry evaluation.

### Statistical analysis

Values are expressed as mean ± SEM or mean ± SEM for control and experimental samples and statistical analysis were performed by Student’s t test with GraphPad Prism software 5.1. The values were considered statistically significant, if the p value was 0.05 or less.

## Results

### General observations

**Figure 2:**
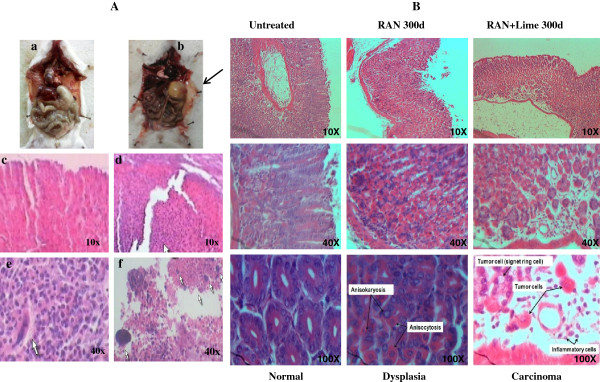
**Dissected mouse and histopathology of both normal and tumor tissue of stomach following treatment with RAN with and without lime. A**. Dissected mouse with (**a**) normal stomach and (**b**) tumorous stomach indicated with an arrow. Histopathology of normal (**c**) and tumour stomach (d,e and f) that induced by RAN extract with lime. The arrows indicate ulcerated neoplasm in (**d**) and tumor giant cells in (**e** and **f**). The magnification is indicated either 10X or 40X. **B**. Histopathology of normal and tumorous stomach of mice following RAN and RAN + lime treatment for 300 days. In all panels, “Normal” indicates mice with no tumor, “Dysplasia” indicates mice with precancerous stomach tissue. “Carcinoma” indicates mice with tumorous stomach. Dysplasia shows anisokaryosis (variation in size of nuclei) and anisocytosis (variation in size of cells). The magnification is indicated either 10X, 40X and 100X.

Out of fifteen mice, two mice developed stomach cancer after 220 and 256 days of feeding of RAN extract with lime (Figure 2A a and b). No tumor was developed in mouse either untreated or administered with RAN only. The histological section clearly differentiated between normal (Figure 2A c) and tumorous stomach (Figure 2A d-f). However, histological preparation of stomach tissue was also made from three apparently normal looking mice randomly selected from this group after 300 days of feeding with RAN-extract with and without lime. Both RAN with and without lime showed its ability to induce cancer in stomach. Two out of three mice treated with only RAN showed pre-cancerous stage (dysplasia), whereas all the three mice treated with RAN + lime showed carcinoma (Figure 2B).

### Studied on metaphase spreads

**Figure 3:**
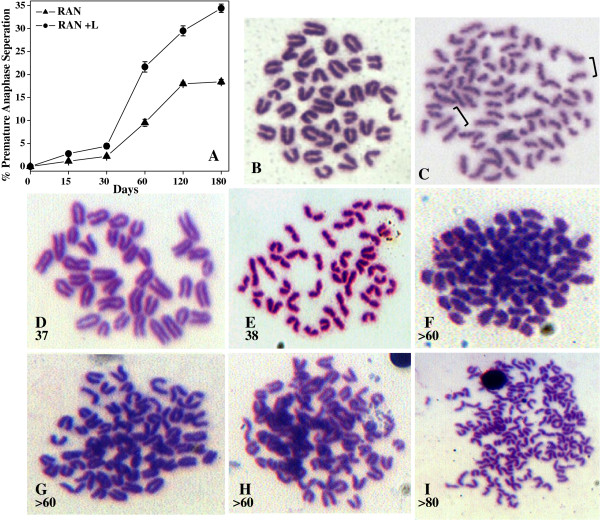
**Karyotype analysis of genomic instability in bone marrow cells of mouse after exposure to RAN extract with (RAN + L) or without lime (RAN). (A)** Percentage of metaphases with premature sister-chromatid separation. Two mice per point for untreated, 15 and 30 days of treated group and three mice per point for the rest. At least 100 metaphases were scored to each mouse. **(B)** Normal metaphase spread from mouse bone marrow cells. **(C)** Premature sister-chromatid separation from mouse exposed to RAN. Brackets show sister-chromatids lying separated in mitotic figures that show the phenotype. **(D and E)** Metaphase spread showed 37 and 38 chromosomes, respectively. **(F**,**G** and **H)** Metaphase spread showed more than 60 chromosomes. **(I)** showed more than 80 chromosomes.

To determine whether continuous administeration of RAN extract (from 15 to 180 days) with or without lime has any effect on chromosomes, we studied metaphase spreads, after 2 h treatment with colchicine, in bone marrow samples. Data revealed a gradual increase in mitotic figures with prematurely separated sister chromatids (Figure 3A and C) both in RAN and RAN + lime administered mouse, compared with none in untreated mice. It is also clear from the study that RAN + lime administered mouse bone marrow showed significantly higher frequency of such precocious anaphase than only RAN administered (Figure 3A). After 180 days of continuous administeration of RAN + lime, 34.4% precocious anaphase compared with 18.4% (p = 0.002) in RAN-administered mouse BMC were seen.

We counted the number of chromosomes in metaphase spreads to understand the significance of precocious anaphases in relation to chromosome stability. The untreated mice have a stable (2n = 40) karyotype (Figure 3B) and did not show any aneuploid cells. We did observe low frequency of aneuploid cells (Figure 3D-I; Table 1) in RAN-administered, with and without lime, for 120 days and it was noted that the frequency of aneuploid cells was increased gradually. The mean frequency (13.8%) of aneuploid cells was scored in both the stomach tumor bearing mice. Overall, the frequency of aneuploid cells was more following RAN + lime administeration than RAN alone (Table 1).

**Table 1:** **Chromosome analysis of mouse bone marrow cells after exposure to** RAN **extract with or without lime**

**Treatment pattern**	**Treatment days**	**Total spread scored**	**Chromosome no.**							**Aneuploidy**	**Premature sister**
			**37**	**38**	**39**	**40**	**41**	**42**	**>60**	**% (Mean)**	**Chromatid separation %**
Untreated	0	110				110				0	0
		104				104				0	0
RAN	120	105		1	1	103				1.9	17.5
		120	2	1	2	115				4.1	18.7
		105	1	2		102				2.8 (2.9)	18.3 (18.2)
RAN + lime		100	2	2	1	95				5.0	31.7
		105	2	2	3	98				6.6	28.6
		110	3	2	1	103		1		6.4 (6.0)	28.3 (29.5)
											p = 0.015^a^
RAN	180	101	1	2	1	97				3.9	18.0
		124	2	2	1	119				4.0	17.7
		114	3	2	1	108				5.3 (4.4)	19.4 (18.4)
RAN + lime		112	3	3	3	103				8.0	32.6
		105	2	2	1	98		1	1	6.6	34.9
		110	4	2		101		2	1	8.2 (7.6)	35.6 (34.4)
											p = 0.002^a^
RAN + lime	220	232	6	9	12	194	7	2	2	16.4	8.2
(with advanced tumor)	256	234	5	4	7	208	5	2	3	11.1 (13.8)	7.1 (7.6)

^a^ statistically significant in paired t-test; two-tailed p value was shown.

Chromosome aberrations were scored mainly as chromatid breaks. Very low frequency of isochromatid breaks was observed and no exchange aberrations were found. The frequency of chromatid breaks and aberrant metaphases was increased gradually from 60 to 180 days of RAN-administeration with or without lime. The frequency of aberrations was more following RAN + lime administeration than RAN alone. The frequency of aberrations was more in both the advanced stomach tumor bearing mice. (For aberration details, see Additional file 1: Supplementary Information).

### Reduced expression of mitotic and spindle checkpoint genes in RAN-treated mice

**Figure 4:**
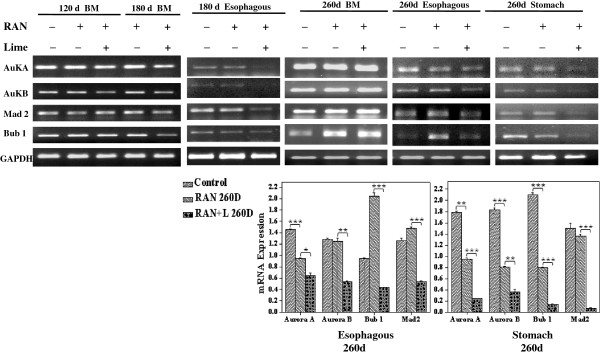
**Upper panel- Expression of mitotic checkpoint genes in mouse bone marrow (BM), esophagus and stomach cells after exposure with RAN extract with or without lime for 120, 180 and 260 days.** Lower panel- Quantitative densitometric analysis of the expression profile of mitotic checkpoint genes mRNA level in esophagus and stomach cells after 260 days of exposure was shown. The values are the mean ± SEM of three independent experiments. The values are normalized to respective GAPDH values. * p < 0.05; ** p < 0.01; *** p < 0.001. Significantly different compared with negative/positive control (as determined by paired t-test).

In view of the above studies, we examined the expression of *AuroraA, AuroraB, MAD2* and *Bub1* genes in bone marrow, esophageal and stomach cells of those mice which were untreated or administered RAN extract with and without lime for 120, 180 and 260 days. The conventional RT-PCR results (Figure 4) showed that cells collected from esophagus and stomach showed mostly under-expression of these genes with respect to untreated one and such under-expression was consistent and significant in RAN + lime administered mice. However, the expression of all these genes in BMC did not change in any significant manner except over-expression of *Mad2* and *Bub1* was noted in the BMC of mouse collected after 260 days of administeration.

### Analysis of over-expression of genes through immunoblotting

**Figure 5:**
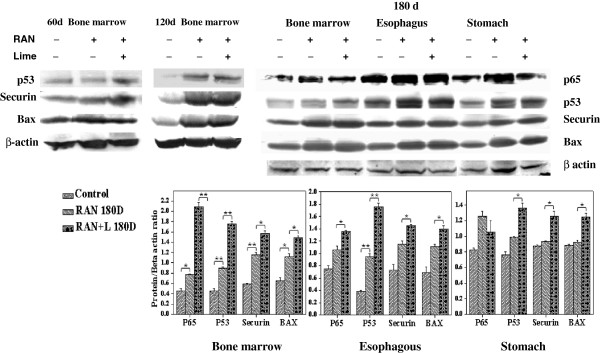
**Upper panel- Representative western blotting detection of p65, p53, securin, bax and β-actin in mouse bone marrow (BM), esophagus and stomach cells after exposure with RAN extract with or without lime.** For BM, cells were collected after 60, 120 and 180 days, whereas for esophagus and stomach, cells were collected only after 180 days of exposure. β-actin was used as loading control. Lower panel- Quantitative densitometric analysis of the level of proteins of the above mentioned genes in bone marrow, esophagus and stomach cells after 180 days of exposure was shown. The values are the mean ± SD of three independent experiments. The values are normalized to respective β-actin values. * p < 0.05; ** p < 0.01 Significantly different compared with negative/positive control (as determined by paired t-test).

Levels of *p53, p65, Bax* and *Securin* in BMC from mice after administeration of RAN extract with or without lime for 60, 90 and 180 days, and those esophagus and stomach after 180 days of feeding were examined by immunoblotting. Levels of these proteins were also tested from the cells collected tissue-wise from the untreated mice. Results indicate that the expression of *p53, p65, Bax* and *Securin* are elevated significantly in all the tissues in RAN administered mice. Such enhancement was significantly higher in RAN + lime than in only RAN administered mice (Figure 5).

### Flow cytometric studies on cell cycle and detection of apoptotic cells by dual staining and immunoblotting

**Figure 6:**
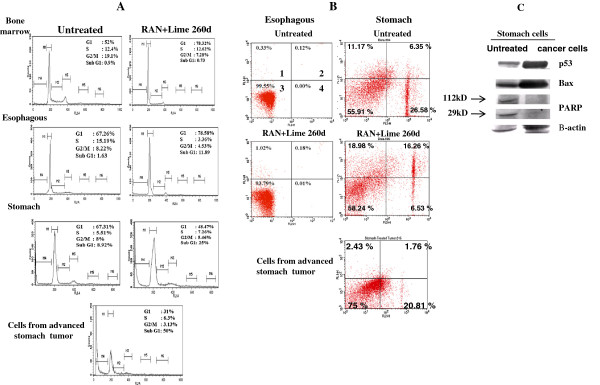
**Analysis of cell cycle and apoptosis in mice treated with RAN with lime (A) Analysis of cell cycle after 260 days of exposure with RAN extract with lime in bone marrow, esophagus and stomach cells of mouse and also from stomach tumor cells. (B)** Representative cytograms of Annexin V versus PI fluorescence intensities as determined by flow cytometric analysis in mouse esophagus and stomach cells after 260 days of exposure with RAN with lime and from stomach tumor cells. Within a cytogram, quadrant 1 and 2 represent early and late apoptotic cells, respectively; quadrant 3, viable cells; quadrant 4, dead cells. **(C)** Western blotting for apoptotic markers in normal (untreated) and tumor stomach cells. β-actin was used as loading control.

Flow cytometric analysis of DNA content in bone marrow, esophagus and stomach cells of mouse collected after 260 days of RAN + lime administration (Figure 6A), showed that there was an increase in G1 phase cells both in bone marrow and esophagus with respect to untreated control. However, in stomach, the percentage of cells in sub-G1 phase was increased, which could be attributed due to apoptotic cell death. To confirm this, dual staining with annexinV and PI was performed. Data in Figure 5B indicate increased positive staining with Annexin-V in quadrant 2 and 3 in stomach cells but not in esophageal cells of RAN + lime administered mice.

Interestingly, flow cytometric analysis of tumor cells collected from the mice which developed tumor in stomach after 220 and 256 days of continuous administeration of RAN and lime, revealed significant reduction in G1 cells with a concomitant rise in the sub-G1 cells (Figure 6A). Dual staining indicates that sub-G1 cells are mostly necrotic dead cells (quadrant 4) (Figure 6B). Significantly higher level of *p53* and *Bax* proteins were observed in tumor than normal stomach cells (Figure 6C). However, *PARP* was found to be absent in both the tumor cells of the stomach although *PARP* and its 29 kD cleaved product were present in normal stomach samples (Figure 6C).

## Discussion

The present study was undertaken to see if *ad libitum* administeration of RAN extract with or without lime in drinking water can induce esophagus or stomach cancer in mouse and if it does, what initial processes are involved. In this study, the mice were given the whole RAN-extract with and without lime in order to mimic the human consumption style of RAN. Moreover, the dose was also increased periodically as it happens to human. Our results showed that both RAN with and without lime induce stomach cancer, although, it is noted that presence of lime with RAN promoted higher cell transformation and thereby developed stomach cancer earlier than RAN alone. It appears that the cancer was induced in stomach because of the greater exposure its lining had to RAN while the esophagus lining was exposed only briefly during drinking the RAN mixed water.

It has been demonstrated earlier that an alkaline pH is ideal for generating ROS by autoxidation of areca-nut polyphenols [6,7]. It was also shown that the catechin fraction of areca-nut extract actively generates ROS at alkaline pH which induces DNA damage *in vitro*[21,22]. Therefore, the yield of ROS in presence of lime could be a contributing factor for the induction of higher cell damages which promoted higher cell transformation in the present case.

The gradual and significant increase in the frequency of precocious anaphase in the BMC of the mouse administered with RAN is interesting. The degree of increase of precocious anaphase was more in the mouse administered with RAN + lime. Such premature sister chromatids separation has been observed in yeast *Mad2* mutants and *Drosophila Bub1* mutants [23,24]. It was demonstrated that partial loss of *Mad2* in Hct 116 cells and in murine primary embryonic fibroblasts showed higher premature sister chromatid separation in the presence of spindle inhibitors and an elevated rate of chromosome mis-segregation events in the absence of these agents [18]. Interestingly, in the present study, such precocious anaphases were observed during early days of exposure which might subsequently lead to production of abnormal cells. Indeed, we observed aneuploid cells in BMC of mouse given RAN with and without lime, initially at low frequency and that was increased gradually irrespective of the development of stomach cancer.

It is likely that the observed precocious anaphase cells lead to chromosome missegregation and subsequent aneuploidy after exposure of 120 days onwards. Such abnormal cells either die apoptotically / necrotically or could be trapped by the cell cycle checkpoints which usually depends on *p53*[25]. In fact, the present flow cytometric analysis of bone marrow and esophageal cells of mouse collected after 260 days of RAN + lime exposure showed that the cell cycle progression is arrested at G1 phase with upregulated expression of p53 protein. However, these cells do not show any apoptosis as revealed from the present flow cytometry studies. In contrast, G1 arrest was not observed in stomach cells, rather sub-G1 phase cells were more frequent which could be a mixture of apoptotic as well as necrotic dead cells. To obtain additional evidence for apoptosis, we tested whether the dying cells exhibited other characteristics of programmed cell death. Immunoblotting demonstrated that RAN with or without lime caused *p53* accumulation and activation of downstream proapoptotic gene like *Bax* which culminated in apoptotic cell death in non-cancerous stomach cells. On the other hand, cytograms of Annexin V versus PI fluorescence intensities for stomach cancer tissue revealed absence of apoptotic cells in spite of a significant rise in the percentage of sub-G1 cells. This suggests, that in cancer tissue of stomach, cells were dying because of necrosis rather than apoptosis.

It is surprising that there was a higher expression of *Bax* while *PARP* was absent in the stomach tumor cells. Apoptotic pathways are considered to be autonomous tumour surveillance mechanisms in a cell whereas evading apoptosis is considered one of the hallmarks of cancer [26]. *Bax* is a pro-apoptotic member of the *Bcl-2* family and is expected to act as tumor suppressor. Therefore, higher expression of *Bax* noted in the present study is unusual. However, there are some earlier reports in which a higher expression of *Bax* in oral squamous cell carcinoma has been noted [27,28]. The absence of *PARP* in the tumor cells of the stomach may be due to aneuploidy in the cancer cells, so that the chromosome on which *PARP* gene resides was lost. Additionally, inhibition or absence of *PARP* has also been noted in several disease models, such as stroke, myocardial infarction, and ischemia [29] in which cells are dying predominantly by programmed necrotic cell death. Further studies are needed to better understand the reasons for absence of *PARP* in the RAN + lime induced stomach cancer cells.

In view of the reports that defective mitotic checkpoint cause chromosomal instability and aneuploidy [23,24], we examined expression of Aurora kinases (*Aurora-A and Aurora-B), Mad2* and *Bub1* in bone marrow, esophagus and stomach cells of the mouse administered with RBN with or without lime for 260 days. Expression of all these genes was found to be significantly downregulated in stomach and esophageal cells of treated mouse. However, the degree of downregulation was more in RAN + lime treated mice. Over-expression of *Mad2* and *Bub1* was noted in BMC of the treated mice. It has been reported that arecoline, a component of areca-nut, upregulated the spindle assembly checkpoint genes like *Aurora A, BubR1* and *Mps1* which led to distorted organization of mitotic spindles and misalignment of chromosomes [30]. The silencing of *Aurora B* by RNA-mediated interference leads to abnormal chromosome segregation and multinucleated cells as a consequence of cytokinesis failure [31]. Reduced *Bub1* expression has been detected in a subset of lung, colon and pancreatic cancers [32,33]. Insufficiency of *Bub1* increased cancer risk in mouse which showed higher frequency of aneuploid cells [34]. The alterations in the expression of these mitotic check-point genes observed in the present study also thus appears to play a significant role in premature sister-chromatid separation followed by chromosome mis-segregation.

*Securin*, also known as pituitary tumor transforming gene, is a key mitotic check-point protein involved at the metaphase-anaphase interface. *Securin*, which is involved in chromatid separation, has transforming activity in vitro and is over expressed in many tumors [35,36]. Over-expression of *Securin* gene in bone marrow, esophagus and stomach cells was noted even during early days of RAN exposure. Over-expression of *Securin* has been shown to induce aneuploidy, arising from chromatid missegeration in human cell [37]. It has been shown that over-expression of *Securin* inhibited chemical-induced DNA double strand break repair activity by repressing Ku heterodimer function [38]. Therefore, *Securin* over-expression may reflect a greater DNA damage, particularly in stomach which was maximally exposed to RAN extract and lime.

Besides *Securin*, we also observed increased expression of *p53* and *p65 (relA*) in all the tissues of mice that were exposed to RAN-extract with or without lime. It has been demonstrated that genotoxic stress elicits a series of posttranslational modifications on *p53,* which contribute to its stabilization, nuclear accumulation and biochemical activation [14]. Mutation in *p53* gene is known to be associated with a variety of human and experimental animal cancers. The accumulation of *p53* protein or its stabilization in all the RAN treated (with or without lime) cells is an important indicator of the presence of mutant *p53* protein as proposed earlier [39]. The *p65,* which is one of the constituent subunit of hetero- or homo-dimers of *Nuclear factor–kappa B (NF-kB*), acts as a regulator of expression of multiple genes that control cell proliferation and cell survival [40]. Activation of *NF-κB* is frequently seen in tumors and plays a pivotal role in linking inflammation to tumor development and progression [41,42].

Research over the years has generated sufficient evidence to implicate areca-nut, as a carcinogen in humans [43,44]. In addition to oral, significant increase in the incidence of cancers of the esophagus, liver, stomach, pancreas, larynx and lung were seen among areca-nut-chewers [45]. Present study shows that RAN can induce stomach cancer and the development of such cancer will be accelerated if lime combines with RAN. Present study provides some insights into the paths that result in aneuploidy and consequently to cancer following RAN treatment. It seems that CIN is a quantitative trait influenced by many genes. Here we show that *Securin* over-expression even at earlier days can elevate CIN and subsequently under-expression of other mitotic check-point genes and over-expression of *p65* and many other relevant genes may be a likely cause of its oncogenicity. Present study also highlights the importance of cytogenetic marker like- premature sister-chromatid separation, as a screening for identification of mitotic check point defects and can be used in heavy-chewers samples.

## Conclusions

Present study suggested (a) RAN induces stomach cancer, however, presence of lime promoted higher cell transformation and thereby developed cancer earlier, (b) perturbations in components of the chromosome segregation machinery could be involved in the initial process of carcinogenicity and (c) the importance of precocious anaphase as a screening marker for identification of mitotic check point defects during early days.

## Competing interests

The authors declare that they have no competing interests.

## Authors’ contributions

Members listed below made their respective contributions to the manuscript. Prof. AC designed the skeleton of this study, supervised the experimental work, analyzed the data with others and drafted the manuscript. SK, AB and NG carried out the experimental work, performed the statistical analysis and prepared the draft figures and tables. HD performed the histopathological work. All authors read and approved the final manuscript.

## Pre-publication history

The pre-publication history for this paper can be accessed here:

http://www.biomedcentral.com/1471-2407/13/315/prepub

## Supplementary Material

Additional file 1 Supplemental information. Click here for file
