# Supplementary material for: Induction of chromosome instability and stomach cancer by altering the expression pattern of mitotic checkpoint genes in mice exposed to areca-nut
Source: BMC Cancer. 2013 Jun 28;13:315. doi: 10.1186/1471-2407-13-315 (PMC3727982; doi:10.1186/1471-2407-13-315)
Supplement: Additional file 1 — Supplemental information. [file 1471-2407-13-315-S1.doc]

**Supplemental Information**

Extended Experimental Procedures

*Scoring of chromosomal aberrations*

Chromosome aberrations were scored as isochromatid breaks (both terminal and interstitial) and chromatid breaks. Exchange aberrations were not found. Translocations were not scored since reciprocal translocation cannot be distinguished without G-banding.

*Primer sequences*

Both forward and reverse primers of Aurora A, Aurora B, Bub1, Mad2 and GAPDH genes were used in conventional RT-PCR analysis. The sequences of the primers are mentioned below. These PCR primers for the specific genes were designed using primer design software Primer3 and verified for specificity using BLAST software.

AURKA (Forwad) : ACATCCTCAGGCTGTATGGCTATTT Exon 6 (665-689)

(Reverse): CCGTTTGAGCCAAGCAGTAAGTTCT Exon 7 (893-869)

AURKB (Forward): TGGAGAATGGCTCAGAAGGAG Exon 1 (313-333)

(Reverse): TGTTGGGATGTTTCAGGTGCG Exon 4/5 (742-722)

Bub1 (Forward): TGTCCTGAGATGCTCAGTAAC Exon 23 (2950-2970)

(Reverse): AGTGTGTTTTGGAGAAGGTCC Exon 25 (3321-3301)

Mad2 (Forward): GCCGAGTTTTTCTCATTTGG Exon 1/2 (120-139)

(Reverse): TTTGAGATGACCACCACCAG Exon 3 (328-309)

GAPDH (Forward): ACAGTCCATGCCATCACTGCC Exon 5 (573-593)

(Reverse): GCCTGCTTCACCACCTTCTTG Exon 6 (834-818)

Results

*Chromosome aberrations*

The frequency of isochromatid breaks was very low. Therefore, the frequencies of aberrant metaphases (Fig. S1 A) and chromatid breaks (Fig. S1 B) were shown. Dose dependent increase in the frequency of aberrant metaphases and chromatid breaks is visible. The overall frequency of aberrations was more in raw areca-nut and lime (RAN+lime) treated mice.


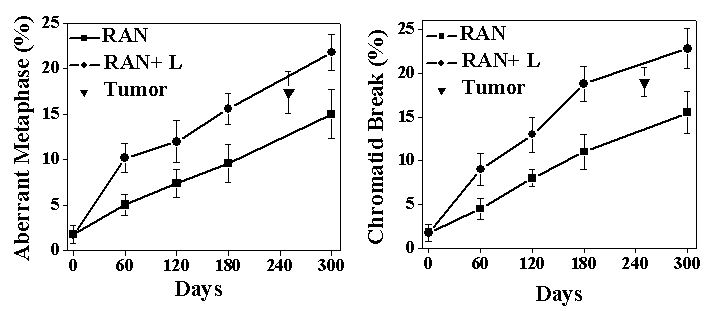


**A** **Fig. S1** **B**

**Figure S1**. Percentage of aberrant metaphases (A) and chromatid break (B) scored in bone marrow cells of mice after exposure to RAN extract with lime (RAN+L) or without lime (RAN). At least 100 metaphases were scored to each mouse. Values are from three separate experiments and expressed as mean ± SD for control and experimental samples. The mean

values of two tumour mice also showed with a different symbol.
